# Regional Brain Glucose Hypometabolism in Young Women with Polycystic Ovary Syndrome: Possible Link to Mild Insulin Resistance

**DOI:** 10.1371/journal.pone.0144116

**Published:** 2015-12-09

**Authors:** Christian-Alexandre Castellano, Jean-Patrice Baillargeon, Scott Nugent, Sébastien Tremblay, Mélanie Fortier, Hélène Imbeault, Julie Duval, Stephen C. Cunnane

**Affiliations:** 1 Research Centre on Aging, Sherbrooke University Geriatrics Institute, Sherbrooke, QC, Canada; 2 Health and Social Sciences Center–Sherbrooke University Geriatrics Institute, Sherbrooke, QC, Canada; 3 Department of Pharmacology and Physiology, Université de Sherbrooke, Sherbrooke, QC, Canada; 4 Department of Medicine, Université de Sherbrooke, Sherbrooke, QC, Canada; 5 Research Center of the Centre hospitalier universitaire de Sherbrooke, Sherbrooke, QC, Canada; 6 Sherbrooke Molecular Imaging Center, Université de Sherbrooke, Sherbrooke, QC, Canada; 7 Department of Nuclear Medicine and Radiobiology, Université de Sherbrooke, Sherbrooke, QC, Canada; 8 Department of Neurology, Centre hospitalier universitaire de Sherbrooke, Sherbrooke, QC, Canada; Peking University Third Hospital, CHINA

## Abstract

**Objective:**

To investigate whether cerebral metabolic rate of glucose (CMR_glu_) is altered in normal weight young women with polycystic ovary syndrome (PCOS) who exhibit mild insulin resistance.

**Materials and methods:**

Seven women with PCOS were compared to eleven healthy female controls of similar age, education and body mass index. Regional brain glucose uptake was quantified using FDG with dynamic positron emission tomography and magnetic resonance imaging, and its potential relationship with insulin resistance assessed using the updated homeostasis model assessment (HOMA2-IR). A battery of cognitive tests was administered to evaluate working memory, attention and executive function.

**Results:**

The PCOS group had 10% higher fasting glucose and 40% higher HOMA2-IR (*p* ≤ *0*.*035*) compared to the Controls. The PCOS group had 9–14% lower CMR_glu_ in specific regions of the frontal, parietal and temporal cortices (*p ≤ 0*.*018*). A significant negative relation was found between the CMR_glu_ and HOMA2-IR mainly in the frontal, parietal and temporal cortices as well as in the hippocampus and the amygdala (*p* ≤ 0.05). Globally, cognitive performance was normal in both groups but scores on the PASAT test of working memory tended to be low in the PCOS group.

**Conclusions:**

The PCOS group exhibited a pattern of low regional CMR_glu_ that correlated inversely with HOMA2-IR in several brain regions and which resembled the pattern seen in aging and early Alzheimer’s disease. These results suggest that a direct association between mild insulin resistance and brain glucose hypometabolism independent of overweight or obesity can exist in young adults in their 20s. Further investigation of the influence of insulin resistance on brain glucose metabolism and cognition in younger and middle-aged adults is warranted.

## Introduction

There is growing experimental and clinical evidence that in addition to controlling glucose homeostasis in peripheral tissues such as liver, muscle and adipose tissue, insulin modulates brain function and energy metabolism [1]. In humans, a close relationship has been shown between plasma insulin and brain glucose uptake using positron emission tomography (PET) with the glucose analog, ^18^F-flurodeoxyglucose (FDG) [2,3,4]. The brain's high energy requirement makes it vulnerable to impaired brain glucose uptake or metabolism, so it is not surprising that insulin/glucose dysregulation could be associated with cognitive impairment [5]. Indeed, insulin resistance, type 2 diabetes and changes in brain glucose metabolism are emerging as central factors in the pathogenesis of Alzheimer’s disease (AD) [6]. Craft’s group reported an AD-like pattern of brain glucose hypometabolism in cognitively normal 74 year olds with pre-diabetes or type 2 diabetes [7]. A recent study reported that insulin resistance measured by the homeostasis model assessment (HOMA-IR) was associated with reduced brain glucose metabolism at 60 years old [8]. Indeed, insulin resistance [9] and regional brain glucose hypometabolism [10] are also common during normal aging.

Our aim in the present study was to assess a condition in which the possible influence of mild insulin resistance on brain glucose metabolism could be studied independently of aging and obesity. Polycystic ovary syndrome (PCOS) is an endocrine disorder involving infertility, hyperandrogenism, and insulin resistance in women [11,12]. Many but not all women with PCOS are overweight. Normal weight PCOS is therefore a model of insulin resistance in a younger adult population that is distinct from the confounding effects of aging and obesity. The primary aim of the present study was to determine whether normal weight women with PCOS had altered cerebral metabolic rate of glucose (CMR_glu_) in gray matter of brain regions associated with AD. The secondary aims were to determine whether differences in CMR_glu_ in PCOS were associated with insulin resistance, or altered cognitive performance. PCOS is commonly associated with higher body-mass index (BMI) but we specifically selected cases with normal BMI in order to reduce the potential confounding effect of higher body weight on CMR_glu_ [13].

## Materials and Methods

### Participants

PCOS (n = 7) was diagnosed during a clinical examination using the Rotterdam criteria [14]. Both the Control (n = 11) and PCOS groups had similar age, anthropometry and metabolic parameters and differed only in that the PCOS group had 10% higher fasting glucose and 40% higher HOMA2-IR (Table 1). None of the participants (PCOS or Control) had diabetes or was taking insulin-sensitizing drugs or medications for diabetes. All participants were current users of oral contraceptives. Exclusion criteria included drug addictions, major depression, psychiatric illness, smoking, or overt evidence of heart, liver or renal disease. This study was conducted with the written informed consent of all the participants and was approved by the appropriate ethics committees (Health and Social Services Center–Sherbrooke University Geriatrics Institute and the Centre hospitalier universitaire de Sherbrooke). This study is registered at ClinicalTrials.gov with identification number NCT02409914.

**Table 1:** Characteristics of healthy age-matched young women (Control, n = 11) *versus* women with polycystic ovary syndrome (PCOS, n = 7).

Characteristics	Control	PCOS	*p-value*
Age, *years*	24.0 ± 3.3	24.6 ± 5.9	*0*.*430*
Education	16 ± 1	15 ± 1	*0*.*020* ^*a*^
Body Mass Index	23.6 ± 3.0	24.5 ± 2.4	*0*.*188*
Hemoglobin A1c, *%*	5.2 ± 0.2	5.1 ± 0.2	*0*.*263*
Fasting glucose, *mM*	4.1 ± 0.4	4.5 ± 0.3	*0*.*010* ^*a*^
Fasting insulin, *IU/L*	3.9 ± 2.0	5.0 ± 2.3	*0*.*158*
HOMA2-IR	0.5 ± 0.3	0.7 ± 0.3	*0*.*035* ^*a*^
Lactate, *mM*	1.3 ± 0.3	1.1 ± 0.2	*0*.*182*
Cholesterol, *mM*	4.5 ± 0.9	4.3 ± 1.0	*0*.*238*
Triglycerides, *mM*	0.9 ± 0.5	0.7 ± 0.2	*0*.*407*
Free fatty acids, *mM*	1.0 ± 0.3	0.9 ± 0.2	*0*.*182*
Creatinine, *μmol/L*	63 ± 10	72 ± 15	*0*.*189*
Thyroid stimulating hormone, *mUI/L*	2.2 ± 0.5	3.3 ± 2.7	*0*.*176*
Testosterone (nmol/L)	≤ 0.5	1.9 ± 0.6	*< 0*.*01* ^*a*^

HOMA2-IR: calculated insulin resistance based on homeostatic model assessment [19].

^a^ *p* ≤ 0.05.

### Cerebral brain glucose consumption with neuroimaging

For each participant, brain positron emission tomography (PET) images were obtained on a PET/CT scanner (Gemini TF, Philips Medical System, Eindhoven, The Netherlands). Briefly, after a fasting period of 6–7 h, each participant was positioned in the PET-scanner in the early afternoon in a dark quiet environment. After intravenous administration of 5.6 ± 0.6 mCuries of FDG via a forearm vein catheter, dynamic scans (field of view = 25 cm, axial field = 18 cm and 2 mm isotropic voxels) were obtained over a total duration of 60 min (time frames = 12 ☓ 10 sec, 8 ☓ 30 s sec, 6 ☓ 4 min, and 3 ☓ 10 min). Several blood samples were obtained to detect FDG radioactivity with a gamma counter (Cobra, Packard, United States) in order to determine the plasma time–activity curves required for the quantification of CMR_glu_ expressed as μmoles/100 g/min. In the regional analysis of CMR_glu_, the brain was segmented into 74 regions as defined by Freesurfer parcellation labels (Freesurfer Suite 5.0). CMR_glu_ was calculated according to the Patlak method [15]. All regional analyses of CMR_glu_ were performed using tools implemented in PMOD 3.3 (PMOD Technologies Ltd., Zurich, Switzerland), as previously described [16]. Calculation of CMR_glu_ included a magnetic resonance (MR)-based correction for partial volume effect and differences in brain volume.

### Volumetric magnetic resonance imaging

As previously described [10], T1-weighted brain MR images (TR = 16.00 ms, TE = 4.68 ms, field of view = 256 ☓ 240 ☓192 mm, matrix size = 256 ☓ 256 ☓ 164, flip angle = 20^◦^ and 1 mm isotropic voxels) were obtained for each participant using a 1.5 Tesla scanner (Sonata, Siemens Medical Solutions, Erlangen, Germany). Regional and whole brain volumes were determined using FreeSurfer Suite 5.0 (Martinos Center for Biomedical Imaging, Massachusetts General Hospital, Harvard Medical School, Cambridge, MA, USA). Cortical brain anatomy was automatically parcellated into 33 regions of interest according to the Desikan-Killiany atlas [17]. Subcortical structures were segmented into 40 regions of interest [18].

### Biochemical analysis

Most plasma parameters were measured using an automated clinical chemistry analyzer (Dimension Xpand Plus; Siemens Healthcare Diagnostics, Deerfield, IL, USA). Plasma total testosterone which was assayed by radioimmunoassay (Diagnostic Systems Laboratories, Webster, TX). Plasma insulin was analyzed by commercial ultra-sensitive enzyme-linked immunosorbent assay (Alpco, Salem, NH, USA) with a Victor X4 multi-label plate reader (Perkin Elmer, Woodbridge, ON, Canada). The homeostasis model assessment computational method was used to estimate insulin resistance (HOMA2-IR) from fasting plasma glucose and insulin [19].

### Cognitive tests

Evaluation of working memory and attention was based on performance on the Paced Auditory Serial Addition Test (PASAT) [20] and the Verbal Digit Span from the Wechsler Adult Intelligence Scale (WAIS-III) [21]. The Trail Making and Stroop Color-Word interference tests from the Delis–Kaplan Executive Function System (D-KEFS), and the Digit Symbol Substitution tests from the WAIS-III and Verbal fluency provided information on executive functions and processing speed [21,22,23]. The 16-item Free and Cued Recall (RL/RI-16) is an episodic memory test [24] providing information about the mechanisms of encoding and retrieval/consolidation processes. Cognitive impairment was defined as score more than 1.65 standard deviations (SD) below the normative values for age and education [25]. Composite Z-scores for each cognitive domain were calculated as the mean of all Z-scores for each individual within cognitive domain.

### Statistical methods

Data are presented as mean ± SD. All statistical analyses were carried out using SPSS 17.0 software (SPSS Inc, Chicago, IL, USA). The Mann–Whitney U-test was used for comparisons between the two groups with a statistical threshold of *p*≤0.05. All regional imaging data including CMR_glu_ comparisons underwent a *p* ≤ 0.05 false discovery rate (FDR) correction for multiple comparisons. Linear regression modeling was used to test whether HOMA2-IR was associated with CMR_glu_.

## Results

Fasting glucose and HOMA2-IR were significantly higher in the PCOS group compared to Controls (*p* ≤ *0*.*035*; Table 1). The PCOS group also had about 4 times higher plasma testosterone than normal and one year less education than the Controls (*p* ≤ *0*.*02*).

### Cerebral glucose consumption (CMR_glu_)

**Fig 1:**
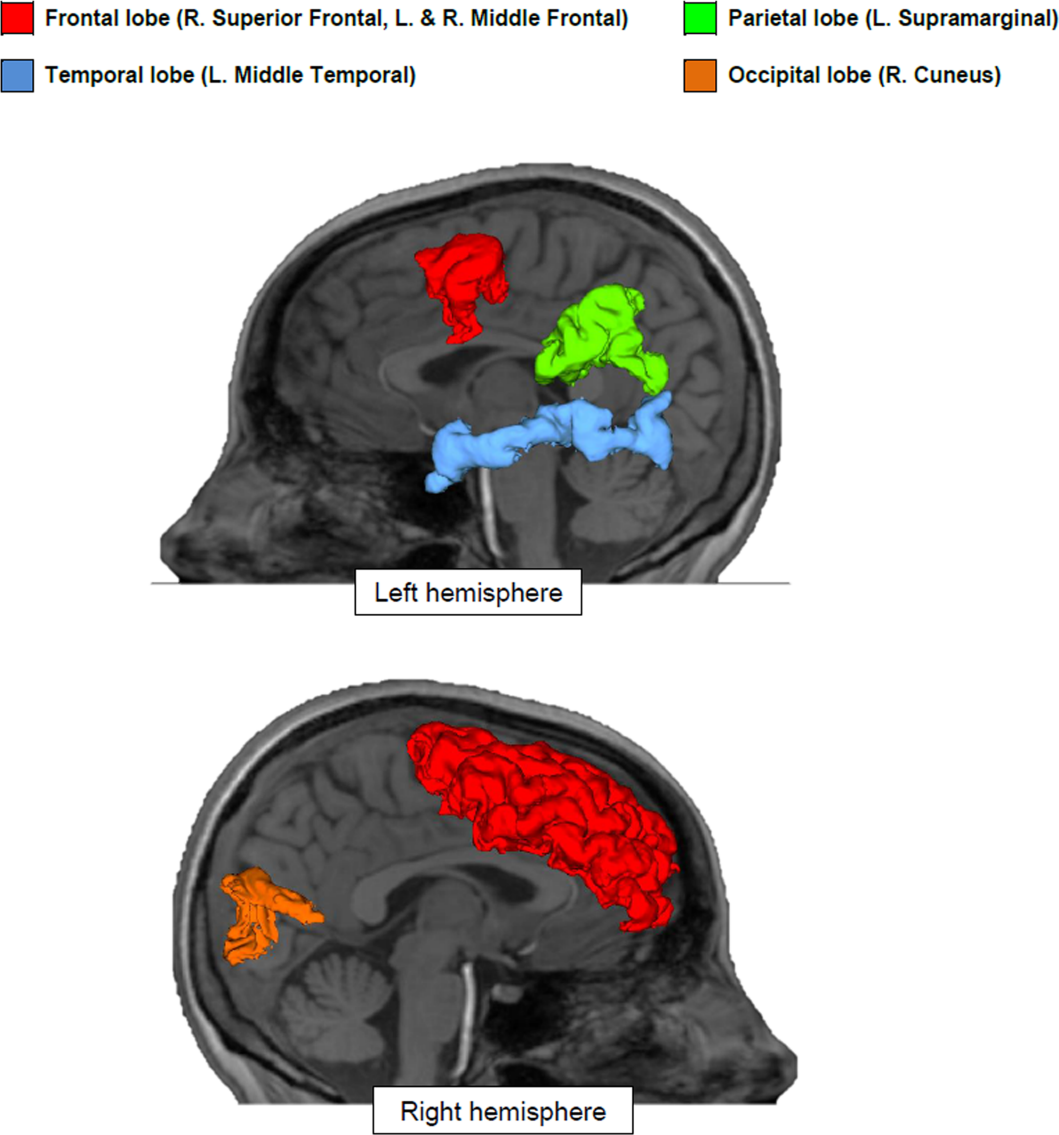
Brain regions with significantly lower glucose uptake in the PCOS compared to the Control group. All results were FDR-corrected for multiple comparisons (*p* ≤ 0.05).

Compared to Controls, CMR_glu_ in gray matter as a whole was not significantly different but tended to be lower in the PCOS group (34.9 ± 3.0 *vs*. 38.0 ± 4.4 μmol/100 g/min; *p* = 0.090, FDR-corrected). In the regional analysis, the PCOS group had significantly lower CMR_glu_ in the right superior frontal cortex (-12%; *p* = 0.018), left middle frontal cortex (-12%; *p* = 0.006), right middle frontal cortex (-13%; *p* = 0.018), left supramarginal cortex (-12%; *p* = 0.006) and left middle temporal cortex (-9%; *p* = 0.006) (Table 2; Fig 1).

**Table 2:** Cerebral metabolic rate of glucose (μmol/100 g/min; mean ± SD) in selected brain regions in healthy young women (Control, n = 11) and women with polycystic ovary syndrome (PCOS, n = 7).

Regions	Left hemisphere		Right hemisphere	
	Control	PCOS	Control	PCOS
*Frontal*				
Superior Frontal	44 ± 6	39 ± 4	44 ± 6	38 ± 2^a^
Middle Frontal	46 ± 5	41 ± 4^a^	46 ± 7	40 ± 3^a^
Orbito-Frontal	40 ± 5	36 ± 6	39 ± 5	36 ± 4
*Parietal*				
Superior Parietal	40 ± 5	36 ± 4	39 ± 4	36 ± 4
Inferior Parietal	41 ± 4	38 ± 4	41 ± 5	37 ± 3
Supramarginal	40 ± 4	36 ± 3^a^	39 ± 5	35 ± 3
*Temporal*				
Superior Temporal	33 ± 3	31 ± 3	35 ± 4	31 ± 3
Middle Temporal	38 ± 4	34 ± 3^a^	39 ± 5	34 ± 2
Inferior Temporal	36 ± 5	33 ± 3	37 ± 5	34 ± 4
Entorhinal	25 ± 3	24 ± 3	27 ± 4	25 ± 3
Parahippocampal	27 ± 3	26 ± 3	28 ± 4	26 ± 2
*Subcortical*				
Thalamus	28 ± 5	27 ± 2	29 ± 4	27 ± 2
Caudate	37 ± 6	33 ± 3	36 ± 5	33 ± 3
Hippocampus	23 ± 2	21 ± 2	23 ± 3	21 ± 1
Amygdala	21 ± 2	19 ± 2	21 ± 3	19 ± 2

^a^Statistically significant difference between groups after p ≤ 0.05 FDR correction for multiple comparisons.

### Regional brain volume and cortical thickness

In comparison to the controls, the PCOS group had 10–17% lower volume of several brain regions, mainly in the frontal and parietal cortex (all *p* ≤ 0.035 FDR-corrected): 10% smaller left superior frontal cortex (22.6 ± 2.0 *vs*. 25.0 ± 2.5 ml; *p* = 0.022), 14% smaller left supramarginal cortex (10.3 ± 1.4 *vs*. 12.0 ± 1.1 ml; *p* = 0.008) and 10% smaller right superior parietal cortex (13.1 ± 1.8 *vs*. 14.7 ± 2.2 ml; *p* = 0.035). Total intracranial volume, ventricle volume and cortical thickness were not different between the two groups (*p* = 0.075, *p* = 0.123, and *p* ≥ 0.105, respectively; FDR-corrected).

### Cognitive profile of the PCOS group

Globally, the PCOS group did not show any clinically significant cognitive impairment, *i*.*e*. no one scored ≥1.65 SD below the normal range for age and education (Table 3). However, on the PASAT, two PCOS participants showed a clinically relevant deficit with a Z-score ± SD of -1.67 ± 0.06, and three others underperformed on this test (Z-score ± SD of -0.89 ± 0.08). On the delayed recall test from the RL/RI-16, five PCOS participants showed a sub-normal score (Z-score ± SD of -1.13 ± 0.61). Composite Z-scores were lower in working memory and episodic memory than in executive functioning (mean composite z-scores ± SD of -0.39 ± 0.47, -0.49 ± 0.39 and +0.38 ±0.52, respectively; Table 3). No correlation was found between insulin resistance (HOMA2-IR) and the different cognitive outcomes (*p* ≥ 0.151; *data not shown*).

**Table 3:** Cognitive test scores (mean ± SD) in women with polycystic ovary syndrome (n = 7) compared to the normal range.

Cognitive tests	Raw score	Z-score
*Attention and working memory*		
PASAT (/60)^a^	52 ± 6	-0.64
Digit span–Forward^b^	6 ± 1	-0.45
Digit span–Backward^b^	5 ± 1	-0.11
Composite Z-score^c^		-0.39
*Executive functions*		
Trail Making—Visual scanning (sec)^d^	19 ± 3	+0.38
Trail Making—Number sequencing (sec)^d^	23 ± 5	+0.67
Trail Making–Letter sequencing (sec)^d^	23 ± 6	+0.57
Trail Making–Number-Letter switching (sec)^d^	50 ± 12	+0.71
Trail Making—Motor speed (sec)^d^	27 ± 6	+0.29
Stroop Color—Word interference test—Naming (sec)^d^	27 ± 7	+0.04
Stroop Color—Word interference test—Reading (sec)^d^	22 ± 7	+0.71
Stroop Color—Word interference test—Inhibition (sec)^d^	43 ± 6	+0.05
Stroop Color—Word interference test–Inhibition- Switching (sec)^d^	53 ± 10	+0.28
Digit symbol substitution test (120 sec)^d^	95 ± 18	+1.14
Verbal fluency—letter "P" (120 sec)^e^	21 ± 5	-0.06
Verbal fluency—letter "R" (120 sec)^e^	17 ± 5	-0.27
Verbal fluency–Animals (120 sec)^e^	32 ± 8	+0.13
Composite Z-score^c^		+0.38
*Episodic memory*		
RL/RI-16—immediate recall (/16)^f^	16 ± 0	0
RL/RI-16—total free recall (/48)^f^	36 ± 2	-0.42
RL/RI-16—delayed recall (/16)^f^	13 ± 2	-0.70
Composite Z-score^c^		-0.49

^*a*^Paced Auditory Serial Addition Test (PASAT) [20].

^*b*^Wechsler Adult Intelligence Scale (WAIS-III) [21].

^c^Average Z-scores across subjects for each cognitive domain.

^d^Delis–Kaplan Executive Function System (D-KEFS) [22].

^e^Cardebat et *al*. 1990 [23].

^f^Van der Linden et *al*. 2004 [24].

### Relationship between CMR_glu_ and HOMA2-IR

**Fig 2:**
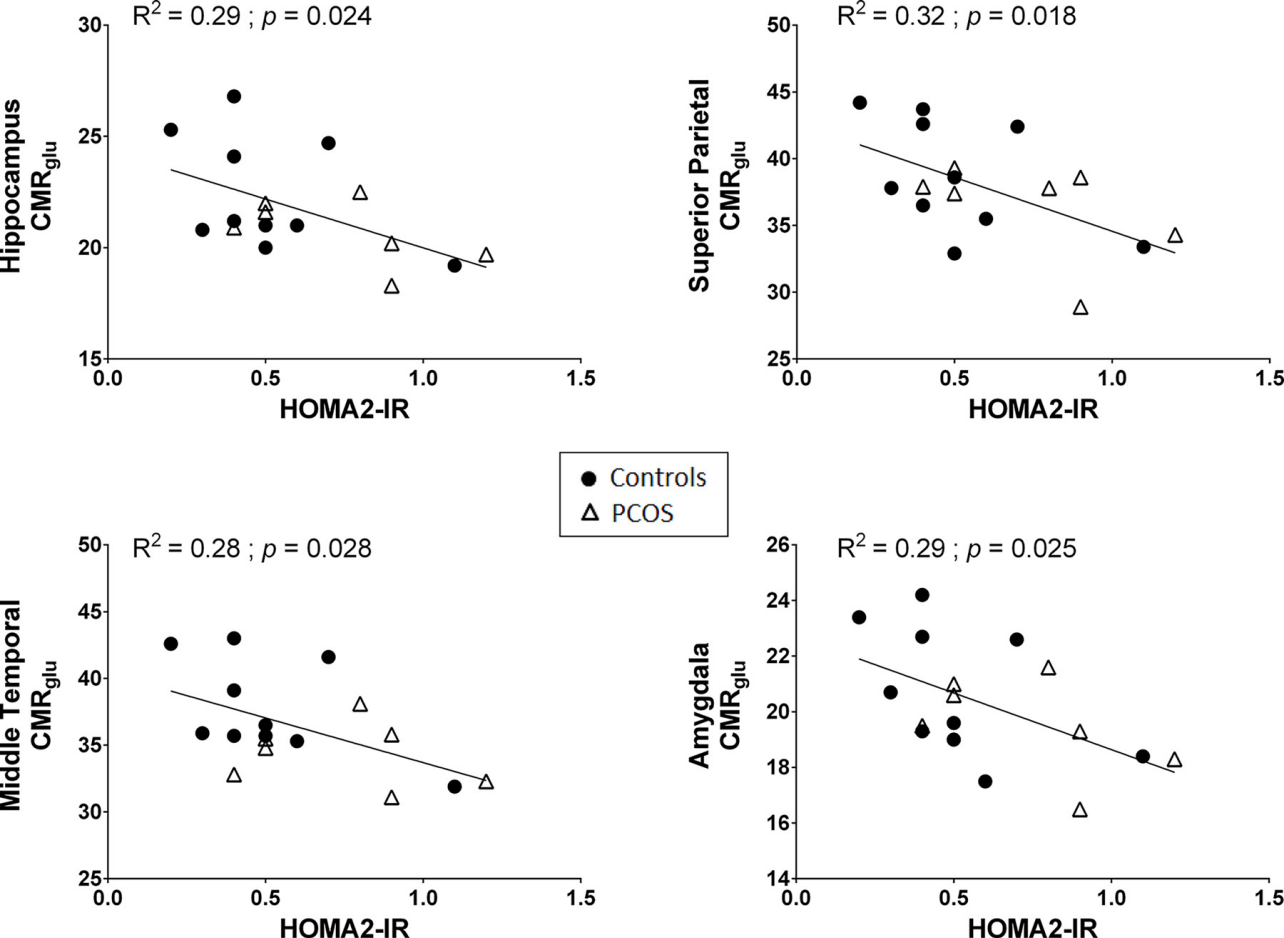
Scatter plot of calculated insulin resistance based on homeostatic model assessment (HOMA2-IR) and average cerebral metabolic rate of glucose (CMR_glu_; μmol/100 g/min) in AD-vulnerable brain regions.

CMR_glu_ in several cortical regions and in the hippocampus and amygdala was significantly inversely related to higher HOMA2-IR (Table 4; Fig 2). Fasting plasma glucose was negatively associated with CMR_glu_ in the middle temporal cortex and caudate nucleus (*p* ≤ 0.03; *data not show*). The potential relationship between plasma total testosterone and CMR_glu_ in the PCOS group was assessed but no significant correlation was found between these two parameters (*p* ≥ 0.150; *data not shown*).

**Table 4:** Relationship between insulin resistance (HOMA2-IR) and cerebral metabolic rate of glucose (CMR_glu_) in the control and PCOS groups combined.

Regions	Left hemisphere			Right hemisphere		
	β	SD	*p-value*	β	SD	*p-value*
*Frontal*						
Superior Frontal	-9.2	3.8	*0*.*027* ^a^	-10.3	3.4	*0*.*009* ^a^
Middle Frontal	-9.0	4.0	*0*.*040* ^a^	-7.6	4.5	*0*.*111*
Frontal Pole	-10.7	4.5	*0*.*031* ^a^	-5.8	7.6	*0*.*454*
*Parietal*						
Superior Parietal	-8.7	3.2	*0*.*016* ^a^	-7.6	3.0	*0*.*022* ^a^
Inferior Parietal	-8.7	2.7	*0*.*006* ^a^	-6.2	3.3	*0*.*081*
Supramarginal	-8.5	2.8	*0*.*008* ^a^	-6.1	3.3	*0*.*089*
*Temporal*						
Superior Temporal	-5.4	2.1	*0*.*020* ^a^	-5.6	3.1	*0*.*096*
Middle Temporal	-6.1	2.6	*0*.*033* ^a^	-7.2	3.4	*0*.*050* ^a^
Inferior Temporal	-5.8	3.3	*0*.*098*	-5.9	4.2	*0*.*181*
Parahippocampal	-5.0	2.5	*0*.*068*	-3.7	3.1	*0*.*252*
Enthorhinal	-3.6	2.4	*0*.*144*	-3.9	3.3	*0*.*245*
*Subcortical*						
Thalamus	-4.1	2.9	*0*.*178*	-4.3	2.9	*0*.*155*
Caudate	-6.8	3.8	*0*.*098*	-4.7	4.1	*0*.*269*
Hippocampus	-4.3	1.6	*0*.*020* ^a^	-4.4	1.9	*0*.*037* ^a^
Amygdala	-3.8	1.5	*0*.*022* ^a^	-4.2	1.9	*0*.*048* ^a^

^a^ *p* ≤ 0.05.

## Discussion

Our main observations are that in comparison with healthy women of similar age, education and BMI, women with PCOS had lower CMR_glu_ in the frontal, parietal and temporal cortex, and that HOMA2-IR was significantly inversely associated with CMR_glu_ in several brain regions. Our findings add young women with PCOS to previous reports of regional glucose hypometabolism in the parietal and temporal regions in middle-aged and older people with insulin resistance as defined by the HOMA-IR [7,8,26,27,28].

Insulin resistance is a well-recognised feature of PCOS [29]. In the present study, the women with PCOS had significant lower insulin sensitivity as determined by the HOMA2-IR. Few human studies have examined brain glucose uptake in an insulin-resistant but non-diabetic population. Higher HOMA-IR was associated with lower CMR_glu_ in frontal, parieto-temporal, and cingulate regions in adults with pre-diabetes; a relationship that was not affected by age [7]. In a large late to middle-aged cohort (61 ± 6 y), Bedlin and collaborators also observed that HOMA-IR was associated with lower brain glucose uptake in the frontal, temporal and parietal regions [27]. In AD, higher HOMA-IR predicts lower brain glucose uptake in the lateral parietal, medial temporal, and prefrontal regions and hippocampus [26]. We observed a significant negative association between fasting glucose and CMR_glu_ in the middle temporal cortex, a region involved in auditory processing and language, and in the caudate, which is part of the motor circuit and is related to body’s voluntary movements. Our results agree with a recent study showing a negative relationship between serum glucose and CMR_glu_ in the primary auditory and motor cortices of non-diabetic young adults aged 28 ± 8 y [30].

Globally, our PCOS group did not show any clinically significant cognitive impairment. However, five of the seven participants underperformed on the PASAT, an indication of potential cognitive difficulties in working memory. Some PCOS participants also had low-normal scores for episodic memory retrieval according to results from the delayed recall test and as confirmed by low-normal composite cognitive Z-scores. Several PET studies in normal aging have reported a relation between deficits in these cognitive domains and functional changes in the prefrontal cortex [31]. The frontal cortex commonly exhibits 10–15% lower CMR_glu_ during aging, a deficit that spreads to the parietal and temporal cortices early in AD [10,16,32,33,34]. Higher HOMA-IR was recently reported to be associated with both lower brain glucose metabolism in left medial temporal lobe and worse memory performance in adults aged 61 ± 6 y [28]. Schattmann and collaborators observed that 28 y old women with PCOS had lower scores in the backward span of the block tapping test, a measure of visuospatial working memory [35]. Women with PCOS may also have impaired performance in other cognitive domains, including reaction time, processing speed [36], visuospatial ability and executive function [37]. Future studies on PCOS should include a cognitive battery that includes an extensive evaluation of attention, working memory and executive function (*e*.*g*. mental rotation test, TAP Divided Attention, etc.) and episodic memory retrieval and consolidation (*e*.*g*. 24 h-delay recall test). More complex tests should be used in order to avoid any potential ceiling effect in episodic memory retrieval. In the present study, no statistically significant relationship was found between insulin resistance and the different cognitive domains evaluated. However, others have found that HOMA-IR is negatively correlated to executive function in healthy women aged 35 [38] and 45 years [39].

The present study was controlled for BMI and age, both of which negatively influence glucose uptake by the brain [40,41,42]. Our dynamic FDG-PET protocol combined with volumetric MRI acquisition and atrophy correction allowed for more accurate quantification of brain glucose uptake [10,43]. Nevertheless, this study had several limitations, notably small sample size and mild insulin resistance, *i*.*e*. all HOMA2-IR scores were <1.5. Although insulin resistance is widely accepted to play a key role in the pathogenesis of PCOS [11,29] and its prevalence is around 64% in women with PCOS [44], our PCOS group was not recruited on the basis of a particular threshold of HOMA2-IR but rather on the basis of being normal weight which is uncommon in PCOS but was important so as to avoid overweight or obesity as a confounder. Fulghesu and collaborators indicated that “a normal HOMA score is not sufficient to exclude early metabolic abnormalities such as hyperinsulinemia in young lean PCOS subjects” [45]. A previous study using the glucose-insulin clamp technique reported lower insulin sensitivity in women with PCOS [46]; in future, this would be the best way to characterize the degree of insulin resistance in women with PCOS. The PCOS group had higher testosterone levels and hyperandrogenism, both of which could potentially modulate both insulin sensitivity [47] and brain glucose metabolism [48]. No correlation was found between plasma total testosterone and CMRglu in the PCOS group but considering our small sample size, we could not adequately assess a potential effect of hypertestosteronemia on brain glucose uptake in this group.

## Conclusions

Our results show that untreated normal weight women with PCOS had lower regional brain glucose uptake in a pattern that resembles that seen in older persons and, to a lesser extent, in early AD. Thus, mild insulin resistance and hyperandrogenism can be associated with regional brain glucose hypometabolism in young adults in their 20s. There was a non-significant trend towards potential cognitive difficulties that specifically involve working memory in the PCOS group. Further investigation and replication of the influence of mild insulin resistance on brain glucose metabolism and cognition in younger and middle-aged adults is warranted.
